# Modelling the impact of COVID-19-related control programme interruptions on progress towards the WHO 2030 target for soil-transmitted helminths

**DOI:** 10.1093/trstmh/traa156

**Published:** 2020-12-14

**Authors:** Veronica Malizia, Federica Giardina, Carolin Vegvari, Sumali Bajaj, Kevin McRae-McKee, Roy M Anderson, Sake J de Vlas, Luc E Coffeng

**Affiliations:** Departmen t of Public Health, Erasmus MC, University Medical Center Rotterdam, Rotterdam, the Netherlands; Departmen t of Public Health, Erasmus MC, University Medical Center Rotterdam, Rotterdam, the Netherlands; London Centre for Neglected Tropical Disease Research, Department of Infectious Disease Epidemiology, Imperial College London, London, UK; Medical Research Council Centre for Global Infectious Disease Analysis, Department of Infectious Disease Epidemiology, School of Public Health, Imperial College London, London, UK; Medical Research Council Centre for Global Infectious Disease Analysis, Department of Infectious Disease Epidemiology, School of Public Health, Imperial College London, London, UK; Department of Infectious Disease Epidemiology, School of Public Health, Imperial College London, London, UK; Medical Research Council Centre for Global Infectious Disease Analysis, Department of Infectious Disease Epidemiology, School of Public Health, Imperial College London, London, UK; Department of Infectious Disease Epidemiology, School of Public Health, Imperial College London, London, UK; London Centre for Neglected Tropical Disease Research, Department of Infectious Disease Epidemiology, Imperial College London, London, UK; Medical Research Council Centre for Global Infectious Disease Analysis, Department of Infectious Disease Epidemiology, School of Public Health, Imperial College London, London, UK; The DeWorm3 Project, Natural History Museum, London, UK; Departmen t of Public Health, Erasmus MC, University Medical Center Rotterdam, Rotterdam, the Netherlands; Departmen t of Public Health, Erasmus MC, University Medical Center Rotterdam, Rotterdam, the Netherlands

**Keywords:** control programmes, COVID-19-related interruption, individual-based models, soil-transmitted helminths, WHO 2030 target

## Abstract

**Background:**

On 1 April 2020, the WHO recommended an interruption of all activities for the control of neglected tropical diseases, including soil-transmitted helminths (STH), in response to the COVID-19 pandemic. This paper investigates the impact of this disruption on the progress towards the WHO 2030 target for STH.

**Methods:**

We used two stochastic individual-based models to simulate the impact of missing one or more preventive chemotherapy (PC) rounds in different endemicity settings. We also investigated the extent to which this impact can be lessened by mitigation strategies, such as semiannual or community-wide PC.

**Results:**

Both models show that without a mitigation strategy, control programmes will catch up by 2030, assuming that coverage is maintained. The catch-up time can be up to 4.5 y after the start of the interruption. Mitigation strategies may reduce this time by up to 2 y and increase the probability of achieving the 2030 target.

**Conclusions:**

Although a PC interruption will only temporarily impact the progress towards the WHO 2030 target, programmes are encouraged to restart as soon as possible to minimise the impact on morbidity. The implementation of suitable mitigation strategies can turn the interruption into an opportunity to accelerate progress towards reaching the target.

## Introduction

Globally, more than 1 billion people in developing countries are estimated to be infected with at least one species of soil-transmitted helminths (STH).^1^ The STH species that mainly affect humans are two species of hookworm (*Necator americanus, Ancylostoma duodenale*), roundworm (*Ascaris lumbricoides*) and whipworm (*Trichuris trichiura*). STH are considered a major cause of morbidity, particularly in children. Common STH-related morbidities are anaemia, growth impairment, respiratory problems and malnutrition due to malabsorption and nutrient loss.^2^ More severe morbidity is usually associated with moderate-to-heavy intensity (M&HI) of parasitic infection.^1^ The control of morbidity drives the definition of the global target set by the WHO for the elimination of STH as a public health problem (EPHP) by 2030. The target is defined as reaching a prevalence of M&HI infections <2% in school-age children (SAC).^3^ The current guidelines provided by the WHO to achieve this goal recommend preventive chemotherapy (PC) for pre-SAC (age 2–5 y) and SAC (age 5–15 y) once per year in moderate endemicity settings (20–50% precontrol prevalence of any intensity of infection in SAC) and twice per year in high endemicity settings (>50%). No PC is recommended in areas with low pre-control STH prevalence (<20%).^3^

On 1 April 2020, the WHO recommended an interruption of all neglected tropical disease control programmes, including STH, in response to the pandemic caused by the novel coronavirus SARS-CoV-2.^4^ With respect to social distancing, the main public health measure taken to contain the spread of SARS-CoV-2, the WHO advised that evaluation activities and PC administration should be postponed until further notice. The impact of this disruption on gains achieved thus far in the control of STH requires investigation. The time it will take control programmes to catch up with past and predicted progress will have implications for the time (delay) and feasibility of reaching the target by 2030. To minimise the losses, suitable mitigation strategies may have to be implemented when programmes resume.

We use two independently developed stochastic individual-based models to simulate the impact of missing or postponing one or more PC rounds on the control of STH in different endemicity settings, defined by the precontrol situation, for all three STH species. We compare the scenario without interruption (baseline scenario) with different ‘interruption scenarios’, which include restarting the treatment after different interruption lengths, with or without plausible mitigation strategies. For each scenario, we express the estimated impact in terms of three measures: (1) the catch-up time, i.e. the time after the interruption required for the M&HI prevalence to catch up with the scenario without interruption; (2) the probability to reach the control target set by the WHO; and (3) the delay, i.e. how much longer it takes to reach the target with respect to the baseline scenario. We investigate the extent to which this impact can be reduced by suitable mitigation strategies.

## Materials and Methods

### Transmission models

We used two stochastic individual-based models independently developed by Erasmus MC (EMC) and Imperial College London (ICL).^5–7^ These models are used to simulate the process of transmission of STH in an age-structured human population through an environmental reservoir of infection (eggs/larvae). Human hosts can be infected and contribute to the reservoir. The life cycle of worms within the human hosts is also modelled. In both models, a single-slide Kato-Katz faecal smear test is simulated, providing egg counts for each individual. Overdispersion of the detected number of eggs (and thus the probability of finding none) is governed by a species-specific parameter. The two models were calibrated to reproduce the same endemicity settings at precontrol level by varying the species-specific parameters regulating the transmission conditions (i.e. the overall exposure rate to central reservoir of infection [EMC model] or basic reproduction number R_0_ [ICL model]), as well as the level of exposure heterogeneity, which indicates the extent of aggregation of worms among hosts. Both models assume an effective treatment coverage of 75% of the target population (pre-SAC and SAC or the whole community) in all simulated scenarios. Individual participation in PC is assumed to be random, meaning that at each round a new random fraction of the population participates. The population is treated with albendazole, which we assume kills 95% of hookworm, 99% of *As. lumbricoides* and 60% of *T. trichiura* adult worms.^8^ A complete description of the parameters used in both models to run simulations is available in Supplementary Table 1. All analyses were performed in accordance with the Policy-Relevant Items for Reporting Models in Epidemiology of Neglected Tropical Diseases (PRIME-NTD) criteria^9^ (Supplementary Table 2).

### Scenarios and mitigation strategies

**Figure 1.**
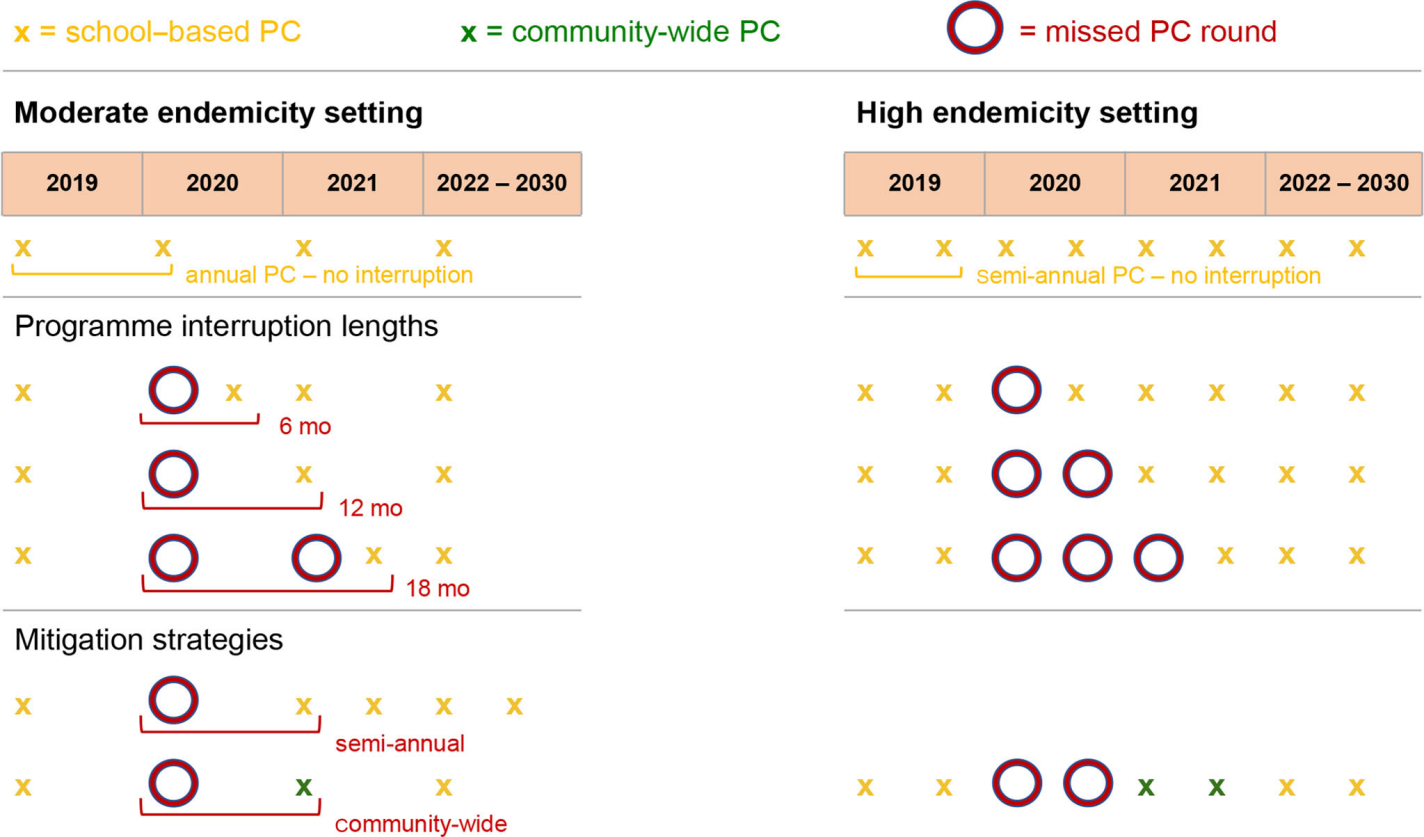
Conceptual diagram explaining the frequency of preventive chemotherapy (PC) for each simulated scenario. School-based PC is distributed once per year in moderate endemicity settings and twice per year in the high endemicity settings. Each cross represents one PC round. Each circle represents one missed round. Each green cross represents one round of community-wide PC. Interruption scenarios without mitigation strategies are presented for different interruption lengths, mitigation strategies are assumed to start 12 mo after the interruption.

Two endemicity settings are considered: namely, moderate transmission (20–50% precontrol prevalence evaluated in SAC by single Kato-Katz) and high transmission (precontrol prevalence >50%). For each species, we ran 500 repeated simulations with both models for each of the six different control scenarios outlined in Figure 1, over a timeline of 12 y (2018–2030), where PC is initiated in 2019. The ‘no-interruption’ scenario (baseline) assumes that no interruption occurs and that PC continues at the same frequency and coverage until 2030. Then we consider that in 2020 a disruption due to the COVID-19 pandemic causes one or more rounds of PC to be missed and that the programme is resumed normally after 6, 12 or 18 mo. As illustrated in Figure 1, according to the endemic setting and the different ‘interruption scenarios’, the number of rounds lost is one to three. To mitigate the potential progress lost during the interruption of PC, we also consider scenarios where programmes are resumed 1 y after the interruption (12-mo interruption), with the addition of the following mitigation strategies: (1) doubling the frequency of PC for the whole period after the interruption; and (2) providing community-wide PC (through mass drug administration [MDA]) for only 1 y after the interruption then going back to treating only pre-SAC and SAC. In the high endemic settings, we do not model the scenario of doubling the frequency of PC, because we consider the administration of four PC rounds per year as unrealistic and logistically infeasible. Also, the additional impact of more than two rounds per year of PC is negligible, given an assumed expected worm’s lifespan of 1 (*As. lumbricoides)*, 1 (*T. trichiura*) or 2–3 (hookworm) y (see Supplementary Table 1).

### Catch-up times and delays

We predict the impact of PC interruption by comparing the dynamics of the prevalence of M&HI infections in SAC in the different simulated scenarios. The results are expressed in terms of three outcome measures: (1) the catch-up time, defined as the time from the interruption until the M&HI prevalence in SAC becomes equal or lower than the one in the scenario without interruption, based on pair-wise differences between single stochastic simulations; (2) the probability of reaching the control target set by the WHO (i.e. the proportion of stochastic simulations showing a M&HI prevalence <2% at time point 2030); and (3) the delay in reaching the target, which is the additional amount of time needed to reach a M&HI prevalence <2% (that remains <2% until 2030) with respect to the scenario without interruptions. The delays are computed based on pair-wise differences between single stochastic simulations that reach the target in the baseline scenario.

## Results

### Interruptions without mitigation strategies catch up by 2030

**Figure 2.**
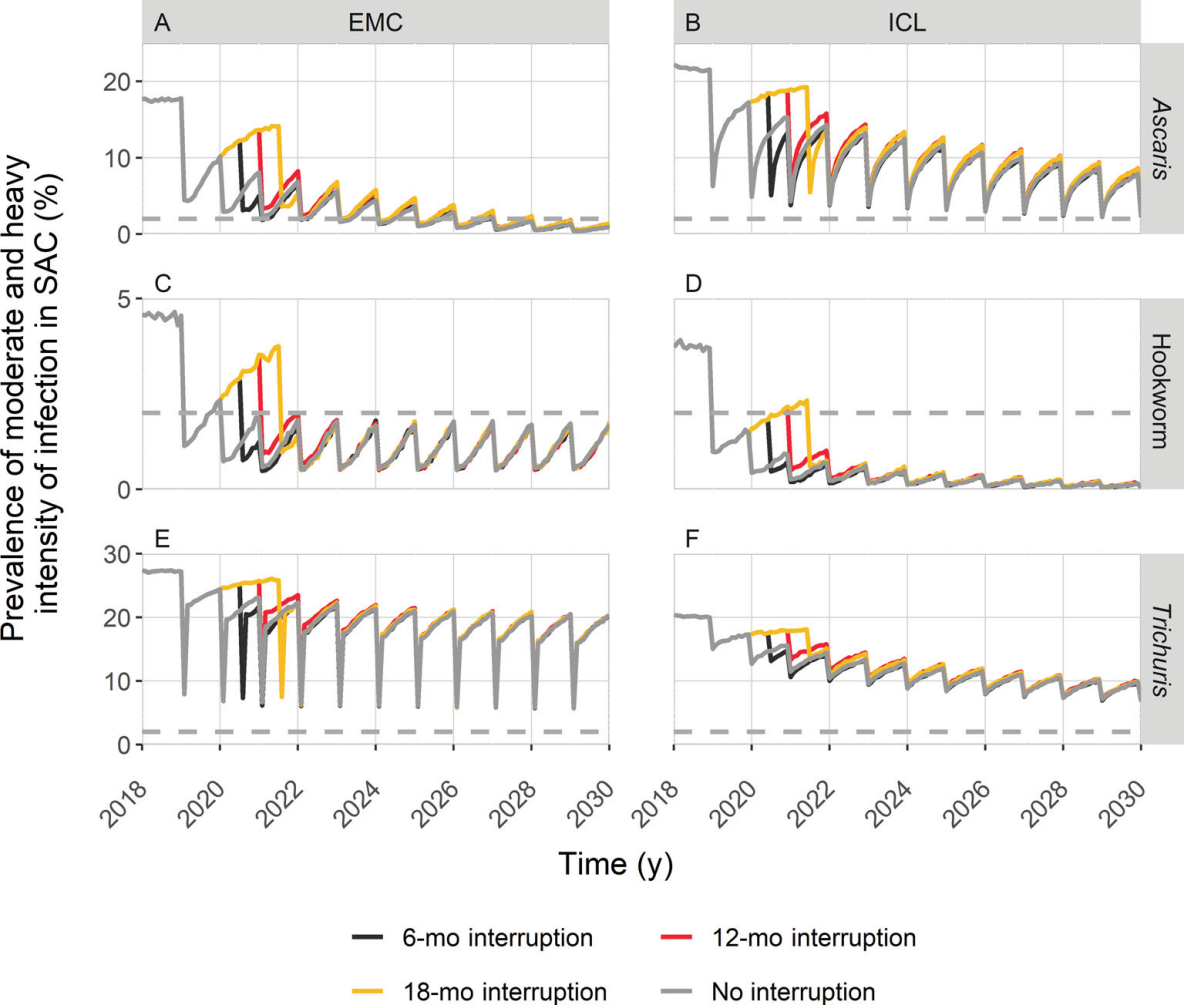
Timeline of moderate-to-heavy intensity infections prevalence in moderate endemicity settings for different interruption lengths if programmes resume without mitigation strategies. The comparison between ‘no interruption’ and restarting after 6, 12 or 18 mo is presented by line colours. The horizontal dashed line represents the 2% threshold set by the WHO to assess the goal. Results from both the EMC model (A, C and E) and the ICL model (B, D and F) are shown, for all STH species.

In Figure 2, we compare the prevalence of M&HI infections in the baseline scenario, i.e. where no interruption occurs, with the interruption scenarios without mitigation strategies, in which up to two PC rounds were missed due to a 6-, 12- or 18-mo programme interruption, starting in 2020. The figure relates to the moderate endemicity setting and it shows that for all species, after interruption, the prevalence eventually catches up with the prevalence of the baseline scenario, according to both models. This means that at some point after the interruption the M&HI prevalence reaches the same value that would have been observed with an absence of interruptions, thus ‘catching up’ with the progress towards the 2030 target that would have otherwise been made. In areas where *As. lumbricoides* is the dominant species it takes slightly longer to recover the progress made by previous control efforts. Supplementary Figure 1 shows the analogous results for the high endemicity setting, where the M&HI prevalence of the baseline scenario is compared with the interruption scenarios without mitigations, in which up to three PC rounds were missed. The figure shows that for all species, the prevalence in the interrupted scenarios catches up with the prevalence of the baseline scenario by 2030. The highest impact of interruption on the M&HI prevalence is observed for *T. trichiura* if an interruption of 12 or 18 mo occurs. Table 1 summarises the impact of programme interruptions in terms of estimated catch-up times, i.e. the average time needed for the M&HI prevalence in SAC in the interruption scenarios to catch up with the baseline scenario. The values are expressed in years from the start of the interruption. In moderate prevalence settings, if programmes resume after 6 mo, the progress in reaching EPHP will be recovered in <2 y (Table 1) and an interruption of 12 mo will require around 3 y in both moderate and high endemicity settings. Interestingly, in moderate endemicity settings, an interruption of 18 mo does not increase the time needed to catch up with the baseline scenario, with respect to an interruption of 12 mo (Table 1). Figure 2 shows that this is due to the beneficial effect of having the first PC round after 18 mo of interruption, which happens only 6 mo before the following normally scheduled round (see Figure 1 for the scheme of scenarios), effectively resulting in 1 y of semiannual treatment. This pattern is not observed in the high endemicity settings because all the scenarios already include semiannual PC (Supplementary Figure 1). In high endemicity settings, the time required for the prevalence to catch up after interruptions of various lengths is limited to on average 2.5 y (hookworm) and on average 3.5 y (*As. lumbricoides*), while *T. trichiura* prevalence requires up to 4.5 y according to both models (Table 1).

**Table 1.** Model-predicted time (mean number of years since start of interruption [95% CI]) required to catch up with the progress towards the 2030 target after 6, 12 or 18 mo of programme interruption compared with a scenario without interruption and assuming that no mitigation strategy is implemented. As moments of catch-up always occur right after a PC round, CIs are presented as integers (in case of annual PC) or multiples of 0.5 (semiannual PC)

		Moderate endemicity setting			High endemicity setting		
Species	Model	6-mo interruption	12-mo interruption	18-mo interruption	6-mo interruption	12-mo interruption	18-mo interruption
*As. lumbricoides*	ICL	1.35 [95% CI 0.5 to 5]	2.89 [95% CI 1 to 8]	2.78 [95% CI 2 to 8]	1.76 [95% CI 0.5 to 4]	2.69 [95% CI 1 to 6]	3.65 [95% CI 2 to 8.5]
	EMC	1.25 [95% CI 1 to 3]	3.56 [95% CI 1 to 9]	2.80 [95% CI 2 to 8]	1.48 [95% CI 0.5 to 4]	2.14 [95% CI 1.5 to 4]	2.67 [95% CI 2 to 5]
Hookworm	ICL	1.20 [95% CI 0.5 to 3]	2.28 [95% CI 1 to 5]	2.35 [95% CI 1.5 to 5]	1.12 [95% CI 0.5 to 3]	1.67 [95% CI 1 to 3.5]	2.15 [95% CI 1.5 to 4]
	EMC	1.29 [95% CI 0.5 to 4]	2.43 [95% CI 1 to 6]	2.47 [95% CI 1.5 to 6]	1.34 [95% CI 0.5 to 3.5]	1.98 [95% CI 1 to 4]	2.54 [95% CI 1.5 to 5]
*T. trichiura*	ICL	1.32 [95% CI 0.5 to 5]	2.26 [95% CI 1 to 5]	2.41 [95% CI 1.5 to 6]	1.87 [95% CI 0.5 to 4.5]	3.27 [95% CI 1.5 to 6]	4.65 [95% CI 2 to 10]
	EMC	1.50 [95% CI 0.5 to 5]	2.85 [95% CI 1 to 8]	2.82 [95% CI 1.5 to 7]	2.02 [95% CI 0.5 to 5]	3.31 [95% CI 1.5 to 7.5]	4.47 [95% CI 2 to 9]

Interruptions up to 18 mo do not have a strong impact on the probability of reaching the WHO target for hookworm, and the time to reach the WHO target will be delayed by between 0.34 (95% CI -1 to 1) and 0.49 (95% CI -2 to 2) (EMC model) and between 0.39 (95% CI -1 to 1) and 1.49 (95% CI 0 to 3) y (ICL model) (Supplementary Table 3). A negative value means that the target is reached earlier than in the baseline scenario. The M&HI prevalence of *As. lumbricoides* is likely to reach levels <2% with 83.2% (416/500) probability in the baseline scenario (EMC) and 26.2% (131/500) (ICL) (Supplementary Table 4). Interruptions do not affect the probability of reaching the target according to the EMC model, but they can delay the time when the target is reached by 0.5 (95% CI -1 to 2) (6-mo interruption) to 1.4 (95% CI 0 to 3) y (18-mo interruption). According to both models, it is not feasible to reach the target by 2030 even without interruptions in settings where *T. trichiura* is the dominant species, due to the relatively low efficacy of albendazole against this STH infection.

### Mitigation strategies help recover and speed up the progress

**Figure 3.**
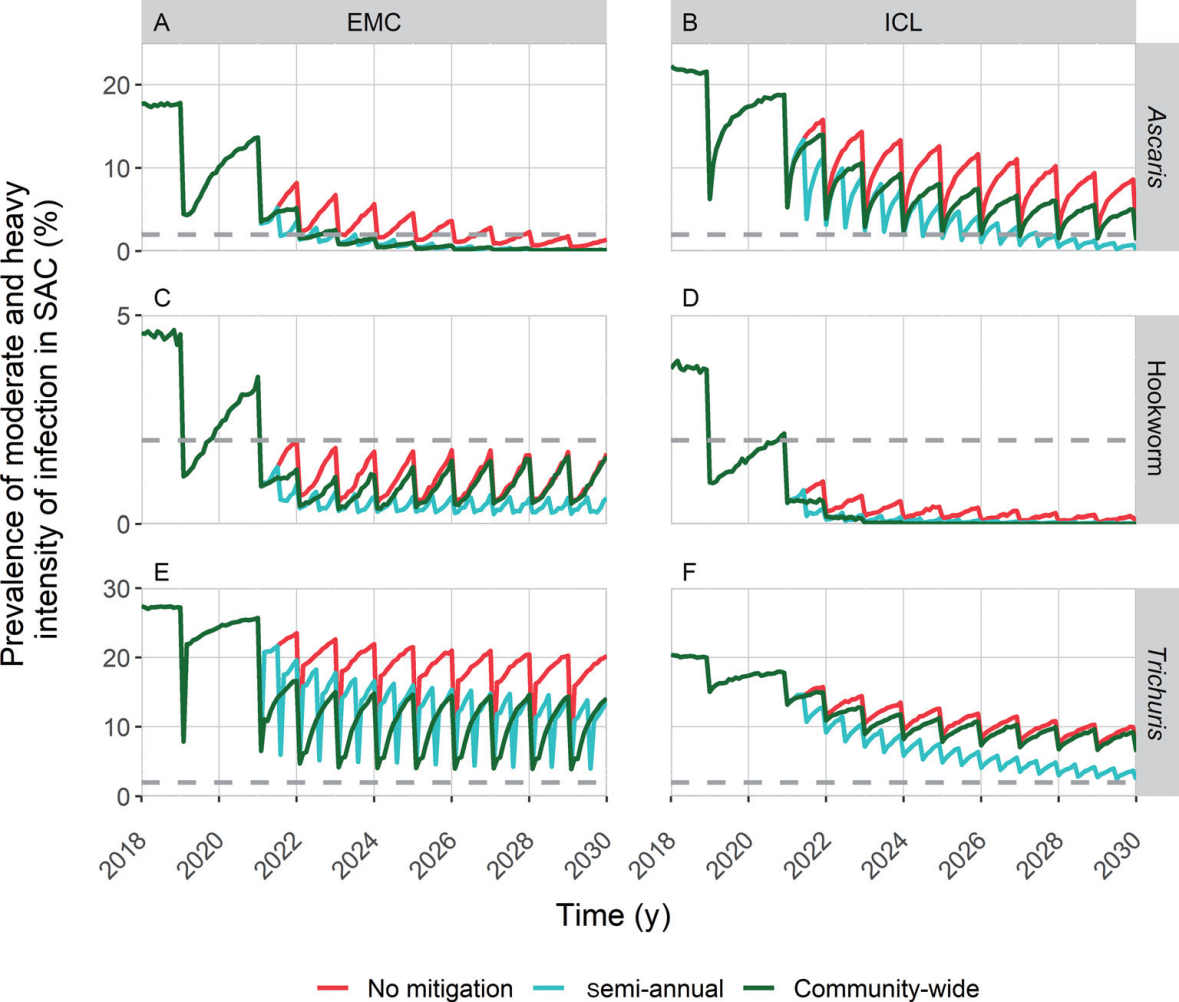
Timeline of moderate-to-heavy intensity infections prevalence in moderate endemicity settings, if programmes resume 1 year after the interruption, (1) without mitigation strategies (red line), (2) doubling the frequency (turquoise line) and (3) providing a first year of community-wide MDA before going back to the current WHO treatment guidelines (dark green line). The horizontal dashed line represents the 2% threshold set by the WHO to assess the goal. Results from both the EMC model (A, C and E) and the ICL model (B, D and F) are shown, for all STH species.

The M&HI prevalence dynamics of the scenarios with two mitigation strategies (semiannual PC and one round of community-wide PC) are compared for the moderate endemic setting in Figure 3. The figure also shows the scenario without any mitigation strategy for comparison. The analogous figure is presented for the high endemic setting in Supplementary Figure 2. We computed the differences between the catch-up time of each mitigation strategy and the catch-up time of the same interruption's length scenario without mitigations. The values are summarised in Table 2. With semiannual PC when resuming programmes in moderate endemic settings, the catch-up will be speeded up by <2 y with respect to resuming without mitigation strategies for *As. lumbricoides*; and by about 1 y in the cases of hookworm and *T. trichiura.* If control programmes implement a 1-y period of community-wide PC then revert to targeting SAC and pre-SAC, about 1 y is gained towards the catch-up for *As. lumbricoides* (both models) and *T. trichiura* (EMC model). In the other cases, the time required to catch up with past progress is not significantly shorter than resuming without mitigation strategies. In high endemic settings, mitigation by means of community-wide PC is beneficial in terms of catch-up time only in the case of *T. trichiura*, allowing prevalence to catch up about 1 y sooner than if no mitigation is applied, for both models (Table 2).

**Table 2.** Model-predicted differences of time (mean number of years [95% CI]) required for each mitigation strategy (semiannual or community-wide preventive chemotherapy [PC]), to catch up with progress towards the 2030 target, compared with the catch-up time required if resuming without mitigation strategies. All values relate to the case of a 1-y programme interruption. As moments of catch-up always occur right after a PC round, CIs are presented as integers (in case of annual PC) or multiples of 0.5 (semiannual PC)

		Moderate endemicity setting		High endemicity setting
Species	Model	Semi-annual PC	Community-wide PC	Community-wide PC
*As. lumbricoides*	ICL	1.26 [95% CI 0 to 6]	0.77 [95% CI -1 to 5]	0.73 [95% CI -1 to 4]
	EMC	1.70 [95% CI 0 to 7]	1.32 [95% CI -1 to 6]	0.39 [95% CI -0.5 to 2.5]
Hookworm	ICL	0.71 [95% CI 0 to 3]	0.35 [95% CI -1 to 3]	0.09 [95% CI -1 to 1.5]
	EMC	0.80 [95% CI 0 to 4]	0.31 [95% CI -1.5 to 3]	0.24 [95% CI -1 to 2]
*T. trichiura*	ICL	0.71 [95% CI -1 to 4]	0.17 [95% CI -2 to 3]	0.82 [95% CI -1 to 4]
	EMC	1.13 [95% CI 0 to 5.5]	1.05 [95% CI 0 to 6]	1.79 [95% CI 0 to 6]

The implementation of mitigation strategies can be essential to enhance the chance to reach the WHO target or to speed up the time to achieve the target. The 66.6% (333/500) probability (EMC model) to reach the target, observed for hookworm in the baseline scenario, increases to 96.6% (483/500) if semiannual PC is implemented when the programmes restart in moderate endemic settings. The achievement of the target can be accelerated up to a mean of 4.8 y. The ICL model generates slightly more pessimistic results in relation to the predicted impact of interruptions in PC on the delay in reaching the WHO targets for hookworm by both mitigation strategies. This is explained by noting that in the absence of interruption (the baseline scenario), the target is reached 0.35 (95% CI 0 to 2) y after the interruption (Supplementary Table 3). Both models agree that the two mitigation strategies can accelerate the moment when the 2030 target is reached, by on average by >2 y for *As. lumbricoide*s, that is, even a single community-wide round is sufficient to compensate for the year missed in moderate endemic settings (Supplementary Table 4). For all three species and for both models, in high endemic settings we are unlikely to reach the EPHP target by 2030 even without interruption, according to the current guidelines (<5% probability), but a single year of community-wide PC (semiannual) when programmes restart will be beneficial for *T. trichiura* in speeding up the progress, according to the EMC model. It shows that in those settings, this mitigation could be essential to increase the probability of reaching the target from 7.8% (34/500) to 71.4% (357/500). The ICL model results are in reasonable agreement in showing the beneficial effect of community-wide mitigation.

## Discussion

The objective of this study was to investigate the impact of disruptions to STH control programmes by the COVID-19 pandemic on the progress towards reaching the 2030 morbidity target, and to estimate to what extent that impact can be reduced by mitigation strategies. Two different, and independently developed, stochastic individual-based models of parasite transmission and PC impact were employed to answer these questions. We assumed that the programmes would resume after varying periods of interruption up to 18 mo in length to account for the uncertainties regarding the duration of the COVID-19 pandemic and the difficulties in resuming control measures. We found that for all STH species, M&HI prevalence catches up before 2030 in both moderate and high endemic settings, even without implementing a mitigation strategy: catch-up times are limited to 1–3 y (moderate endemic setting) and 1–5 y (high endemic setting). Mitigation strategies reduce the catch-up time by <1 y on average and by 2 y at most. In some cases, however, they have the benefit of increasing the probability of reaching the target by 2030, or of reaching it earlier. Thus, mitigation strategies present an opportunity to enhance progress towards EPHP, especially when reaching the target is not feasible by 2030.

Our results further show that it will always be advantageous to recover the last round missed as soon as possible, e.g. 6 mo after the initially planned date. Our analyses show that it is feasible to reach the target in moderate endemic contexts where hookworm or *As. lumbricoides* are the dominant species. Different durations of interruption do not have a strong impact on reaching the target but they can introduce a delay of up to 1.5 (95% CI 0 to 3) y (ICL, hookworm) or of up to 1.4 (95% CI 0 to 3) y (EMC, *As. lumbricoides*).

Although the mitigation strategies considered here have a limited impact on catch-up times, they will help to accelerate the achievement of EPHP by 2030 in moderately *As. lumbricoides*-endemic settings. A single community-wide round is therefore suggested to compensate for a missed year of PC. Mitigation strategies will not help to increase the low probability of reaching the target in the context of *T. trichiura*, but they will enhance progress by lowering the prevalence of M&HI infections. Both models agree that in highly endemic settings for all three species, the target will not be reached by 2030, even without interruption of PC. For *T. trichiura*, this is mainly due to the relatively low efficacy (60%) of albendazole treatment; dual treatment with ivermectin would be required to reach the target.^10^ For high endemic areas, adding a year of semiannual community-wide PC (two rounds) as mitigation proves to be crucial to speed up progress and enhance the probability of reaching the target for *As. lumbricoides* (ICL) and *T. trichiura* (EMC).

The discrepancies observed in the results between the two models can be explained by the different assumptions, such as regarding age patterns in exposure to eggs/larvae in the environment. For instance, the ICL model assumes a flat age profile for hookworm making exposure to the infection independent of age. By contrast, in the EMC model it is assumed that exposure increases during the first 10 y of life then stabilises, such that most of hookworm infection is carried in adults and that a higher worm burden is observed in adults than in SAC. The two different assumptions about age pattern in exposure fit previously published age-intensity profiles observed in the field,^11,12^ and thus potentially reflect different transmission settings. This difference in assumptions is directly reflected in our results: the probability of reaching the morbidity target in areas with moderate prevalence of hookworm infections is higher in the ICL model, given the current school-based treatment guidelines.^7^

COVID-19-related interruptions are affecting several countries with different histories of control. In this study, we decided to implement the first year of treatment for all STH to simulate ongoing PC; the second year is then missed due to COVID-19 preventative measures. We did not investigate the impact of skipping later rounds of PC. However, theoretically missing PC rounds early in the programme would have a more detrimental impact on current progress and achieving goals. As such, our predictions provide a conservative (i.e. pessimistic) foundation upon which to base policy decisions. We further assume that control programmes will restart PC with the same coverage as before the interruption (75% random participation among the target population). However, there may be hurdles in reaching the same participation rates, especially in community-wide PC due to logistics, but also fear or stigma of healthcare workers as an indirect effect of COVID-19. However, we expect that school-based PC will be less affected, provided school attendance returns to pre-COVID rates.

The estimated outcomes of this study can be tested in settings where M&HI prevalence data were collected before the interruption and where collection will continue afterwards. The Geshiyaro project^13^ and DeWorm3,^14^ which focused on the feasibility of interrupting the transmission of parasitic worms by repeated rounds of PC, included STH. In these projects, PC rounds have been delayed by COVID-19, hence they will be a good test of model predictions. Another test of the outcomes proposed with this paper, required in settings where mitigation strategies are implemented, would be to assess if they reached the 2030 target sooner than in settings where mitigation strategies have not been implemented. Testable model outcomes fulfil one of the criteria requested by the PRIME-NTD (Supplementary Table 2).

Overall, we have shown that STH control programmes may not take too long to catch up after the interruption. However, it is important to minimise the catch-up time and to consider mitigation strategies as soon as possible. There is still a paucity of quantifiable evidence and studies to detect direct morbidity from STH, though M&HI infections have been linked to diarrhoea, anaemia, malnutrition and physical and cognitive impairment.^15^ Prolonged intervals without treatment lead to a higher intensity of infection in individuals, increasing the chance of them developing morbidity. At the population level, this is likely to be reflected in an increase in the proportion of individuals with M&HI infections, therefore, a higher prevalence and severity of morbidity. Anaemia, for example, is the main hookworm-related morbidity and it is strongly associated with moderate and heavy hookworm infections. Anthelminthic treatment is an effective means of improving haemoglobin levels.^16^ A consistent drop in haemoglobin level below the WHO threshold defined for anaemia is estimated to occur starting from 2000 eggs per gram, the value defining moderate infections.^17^ It could, therefore, be crucial, especially for children, to prevent prolonged periods with high M&HI.

We show that even a single year of community-wide treatment after the interruption speeds up the progress towards the morbidity target. In some cases, the target may even be reached sooner than without interruption. Even although community-wide treatment will require extending drug donations to adults as well as children, in specific settings which are far from reaching the target, this is not only helpful in speeding up the process, but also offers an opportunity to reach the morbidity target by 2030.

### Conclusions

We estimated that the COVID-19-related interruption of STH control programmes will only temporarily impact the progress towards the EPHP target by 2030, since for all STH species M&HI prevalence after interruption catches up with the prevalence in the scenarios without interruption, recovering the ground lost by 2030. However, after a PC interruption, programmes require a catch-up time (estimated to be <3 y in moderate endemicity settings and <5 y in high endemicity settings), during which endemic areas can attain higher levels of moderate and high infections. We suggest, therefore, to minimise the time without PC and to restart PC as soon as possible, even if that is before the time when the next round of PC would have been scheduled under normal circumstances. In addition, disruption by COVID-19 could be turned into an opportunity to increase the probability of reaching the target in those settings where it is not feasible with the current guidelines, by implementing suitable mitigation strategies. A 1-y period of community-wide treatment after the interruption would speed up the progress towards the morbidity target. In some cases, resuming programmes with a mitigation strategy would present the only possibility of reaching the morbidity target by 2030.

## Supplementary Material

traa156_Supplemental_Files

## Data Availability

The simulations results of this article will be shared upon reasonable request to the corresponding author.
