# Supplementary material for: Modelling the impact of COVID-19-related control programme interruptions on progress towards the WHO 2030 target for soil-transmitted helminths
Source: Trans R Soc Trop Med Hyg. 2020 Dec 14;115(3):253–60. doi: 10.1093/trstmh/traa156 (PMC7798673; doi:10.1093/trstmh/traa156)
Supplement: traa156_Supplemental_Files [file traa156_supplemental_files.zip › Supplementary Table 3.docx]

**Supplementary Table 3.** Time (years since the interruption of the control programme) and probability to reach the 2030 target in the baseline scenario (no interruption); introduced delays (years) in reaching the target for each interruption scenario, compared to the baseline scenario. Hookworm, moderate endemicity setting. As moments when the target is reached are evaluated right after a PC round, confidence intervals are presented as integers (in case of annual PC) or multiples of 0.5 (semi-annual PC).

| **Model** | **Time (mean [95%CI]) and probability to reach the target in the baseline scenario** | **Delay from the baseline scenario**  **(mean [95% CI]) (ys)** | | | | |
| --- | --- | --- | --- | --- | --- | --- |
|  |  | **6-month interruption** | **12-month interruption** | **18-month interruption** | **Semi-annual mitigation** | **Community-wide mitigation** |
| EMC | 8.14 [1; 10]  66.6% | 0.34 [-2; 2] | 0.17 [-2; 2] | 0.49 [-2; 2] | -4.76 [-8; -2] | -1.32 [-3; 0] |
| ICL | 0.35 [0; 2]  100% | 0.39 [-1; 1] | 1.27 [0; 3] | 1.49 [0; 3] | 1.17 [0; 2] | 1.19 [0; 2] |
