# Supplementary figures and images for: Modelling the impact of COVID-19-related control programme interruptions on progress towards the WHO 2030 target for soil-transmitted helminths
Source: Trans R Soc Trop Med Hyg. 2020 Dec 14;115(3):253–60. doi: 10.1093/trstmh/traa156 (PMC7798673; doi:10.1093/trstmh/traa156)

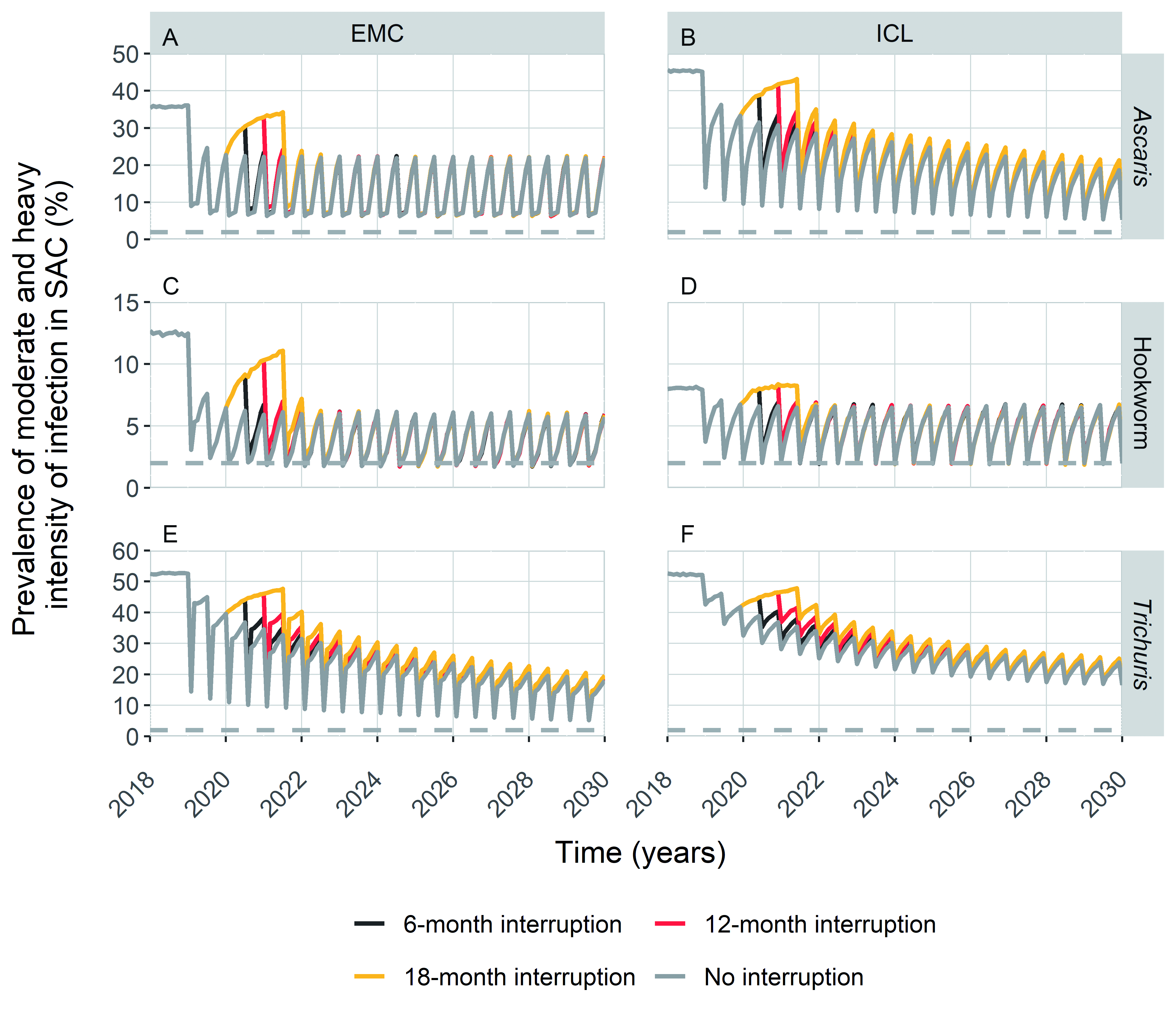

Supplement: traa156_Supplemental_Files [file traa156_supplemental_files.zip › Supplementary Figure 1.tif]

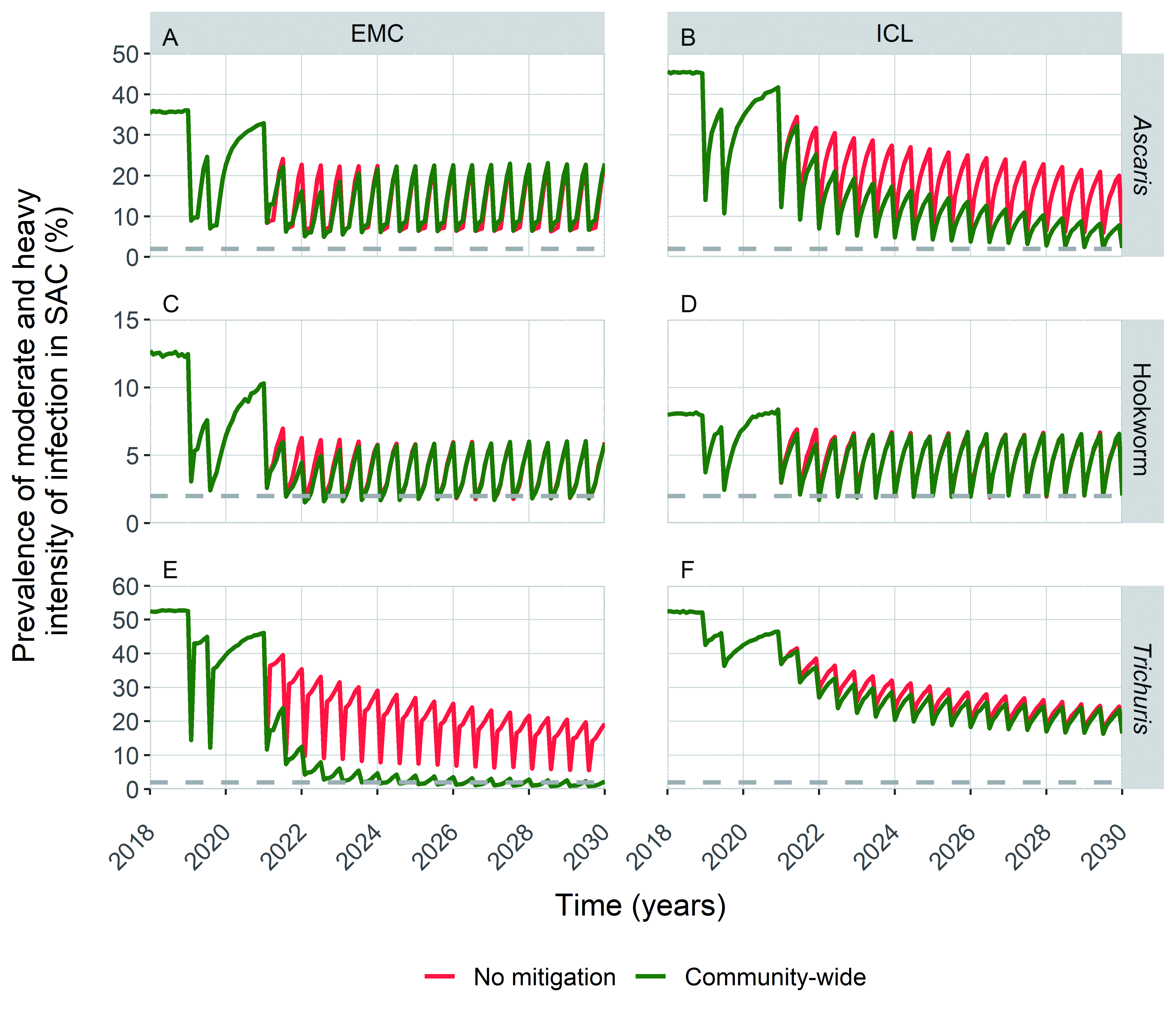

Supplement: traa156_Supplemental_Files [file traa156_supplemental_files.zip › Supplementary Figure 2.tif]
